# Cognitive Function and 30-Day Readmission Risk After Skilled Nursing Facility-to-Home Transitions

**DOI:** 10.1093/geroni/igaf122.3897

**Published:** 2025-12-31

**Authors:** Indrakshi Roy, Jennifer Carnahan, James Slaven, Emily Fox Ludden

**Affiliations:** Indiana University, Bloomington, Bloomington, Indiana, United States; Indiana University Center for Aging Research, Indianapolis, Indiana, United States; Indiana University, Indianapolis, Indiana, United States; Regenstrief Institute, Inc., Indianapolis, Indiana, United States

## Abstract

Transitions from skilled nursing facilities (SNFs) to home are common among Medicare beneficiaries, yet they remain one of the least studied and most vulnerable care transitions. Unlike patients discharged directly home from the hospital, those who first receive SNF care are typically more medically complex, experience multiple transitions, and face a heightened risk of adverse events such as hospital readmissions. Because most SNF patients ultimately return home, understanding this second transition is essential for ensuring quality of care, continuity, and patient and caregiver well-being. We used Health and Retirement Study data linked to Medicare claims and Minimum Data Set (MDS) assessments from 2000–2019 to examine the association between cognitive function and 30-day hospital readmission after SNF-to-home discharge. Cognition was measured using the MDS-based Cognitive Function Scale (CFS). Among 4,527 beneficiaries, we applied generalized estimating equations to generate odds of readmission among those with moderate to severe impairment. After adjusting for demographic and clinical characteristics, CFS was not significantly associated with 30-day readmission (OR = 1.17; 95% CI = 0.91–1.50). However, patients with moderate or severe cognitive impairment had modestly higher unadjusted readmission rates (18.0%) compared with those with intact or mild cognition (15.3%), suggesting unmet needs in discharge readiness or community support. Our findings indicate that cognition alone may not independently predict short-term rehospitalization, but it remains clinically relevant in transitional care planning. By focusing on SNF-to-home discharges, this study highlights a population where targeted interventions—such as enhanced caregiver engagement and community-based services—may reduce preventable readmissions and improve outcomes for older adults.

